# Science, Society, and Dismantling Racism

**DOI:** 10.1089/heq.2022.29023.cro

**Published:** 2023-01-20

**Authors:** Charmaine D.M. Royal

**Affiliations:** Departments of African and African American Studies, Biology, Global Health and Family Medicine and Community Health and Duke Center for Truth, Racial Healing & Transformation, Duke University, Durham, North Carolina, USA.

**Keywords:** race, racism, social hierarchy, genetics, health disparities

## Abstract

As a foundational pillar of the Truth, Racial Healing & Transformation framework, Narrative Change involves reckoning with our historical and current realities regarding “race” and racism, uprooting dominant narratives that normalize injustice and sustain oppression, and advancing narratives that promote equity and collective liberation. Narrative Change is vital to creating communal recognition and appreciation of the interconnectedness and equality of all humans and dismantling the ideology and structures of racial hierarchy. Telling new or more truthful and complete stories must include improving our understanding and messaging about what race is and what it is not as well as the relationship between race and racism. Ideas about the existence of biological human races have long been discredited by scientists and scholars in various fields. Yet, false beliefs about natural and fixed biological differences within the human species persist in some scientific studies, in aspects of health care, and in the political and legal architectures of the United States and other countries, thereby reproducing and maintaining social hierarchies. Efforts to eradicate racism and its pernicious effects are limited in their potential for sustained positive transformation unless simultaneous endeavors are undertaken to reframe people's thinking about the very concept of race. This brief provides an overview of the origins of racial hierarchy, distinguishes between biological concepts of race and socially defined race, reviews perspectives on the meanings and uses of race, and describes ongoing and potential efforts to address prevailing misunderstandings about race and racism.

## The Case for Alternative Narratives

Events in the United States for the past few years have prompted a national and global reckoning with race and racism. The murder of George Floyd, the racial inequities revealed by COVID-19, the hate crimes against individuals and communities identified as Asian, and the mass killing at a Buffalo supermarket all stem from long-standing and deeply embedded racism that permeates our society.^1–4^

The increasing acknowledgment of systemic racism and the apparent mutual desire to address it are heartening. However, our nation has been at similar junctures throughout its history. What will we do differently this time to achieve and sustain the transformation we seek? How will we chart a new path characterized by a richer understanding of and gratitude for our common humanity? When will all lives have equal value and racism no longer shape our individual and collective experiences or outcomes? These questions call for a shift in our approach—from one that focuses almost exclusively on the consequences of racism and racialization to one that devotes adequate attention to both the consequences and the root cause (the underlying belief system) of these inhumane processes and practices. Just as science and society cooperatively invented and perpetuate the illusion of a racial hierarchy they must join forces to right this wrong, generating and circulating counternarratives that will help inform and transform America and the world.

## Race, Racism, and the Ideology of Racial Hierarchy

Race is inextricably associated not only with assumed innate differences between human populations, but also a hierarchy of difference wherein one population is deemed superior to another. It is this twofold formula of racism, not actual biological difference, that shaped ideas about race and racial classifications in humans.^5^ Race, as we have come to understand it, is bound to 15th and 16th century colonial expansions across the world and to the transatlantic slave trade. The ideology of a racial hierarchy, entangled with aspirations for power and domination, was employed to justify the forced colonization of places and populations, the enslavement of Africans, the displacement and genocide of Indigenous peoples, the persecution and massacre of Chinese immigrants, and the Holocaust.^5–7^ The hierarchical thinking that rationalized these atrocities endures today, although in different forms.

During the 18th century, this crude sociopolitical justification for the enslavement and dehumanization of African and other non-European populations was bolstered by the emergent biological sciences of the day in what has become known broadly as race science. However, it is essential to reiterate that it is racism that inspired the taxonomy of race, not vice versa. A system of human taxonomy based on continental origins became part of the scientific lexicon as proposed by Carolus Linnaeus in “Systema Naturae” in 1758 and further cemented by Johann Blumenbach in the 1776 publication “On the Natural Variety of Mankind.”^8–10^ Much of the nomenclature of this time has become obsolete, yet the term ‘Caucasian’ is still widely used; an explicit reminder that while our understanding of human biological variation has been revised, we continue to employ racialized concepts in science and society without reflection on their histories and implications.^11,12^

In addition to Linnaeus and Blumenbach, there were other scientists who both influenced and reflected dominant narratives of the time, especially the notion that populations of European origin were naturally superior intellectually and physically to other populations.^13,14^ However, in the United States, it was Thomas Jefferson, a Founding Father of the newly independent U.S. colonies, who enshrined the place of the descendants of African populations in the newly created nation by stating in his “Notes on the State of Virginia”:

I advance it therefore as a suspicion only, that the blacks, whether originally a distinct race, or made distinct by time and circumstances, are inferior to the whites in the endowments both of body and mind. It is not against experience to suppose, that different species of the same genus, or varieties of the same species, may possess different qualifications.^15^

Notwithstanding Jefferson's pivotal role in the subsequent abolition of the international slave trade, his pronouncements justified continued exploitation and enslavement based on unproven and spurious assumptions about natural biological difference and hierarchy among the so-called human races. The stark contrast between Jefferson's views on race and his views on personal freedom and the right to independent thought and movement is characteristic of the personal and institutional contradictions regarding race during and beyond the founding of the United States. This form of hierarchical racial thinking, combining social and political explanations with race science in or to rationalize enslavement, segregation, and the Jim Crow laws in the United States, continued into the late 19th century up to the mid-20th century.^16–18^

Racial segregation in the United States served as a model for the eugenics movements of that era (aimed at improving the genetic composition of the human species by eliminating characteristics deemed undesirable), the Holocaust, and Apartheid in South Africa.^19–20^ Racial ideologies persist throughout the United States and globally in both crude and overt racial discrimination and more subtle forms of structural racism within institutions and everyday social interactions. As a result of the particular set of historical, economic, and political circumstances from which the North American worldview on race emerged, critical and popular U.S. discourses on race predominantly pivot on a binary black/white axis.^21,22^ However, it is important to note that all people in the United States are racialized, and are granted or denied rights, privileges, and opportunities based on racial classification.

## Biological Versus Social Race

Although the eugenics movement was not universally embraced, it took the horrors of the Nazi regime and the murder of millions of Jews, gypsies, and other “undesirable” people to initiate a widespread and more explicit interrogation or condemnation of the belief in biologically based racial hierarchy.^23^ A new narrative began to emerge, questioning the key scientific assumption that human biological races even exist and the lack of scientific evidence to suggest that racial differences in health, physical features, behaviors, and other characteristics were the product of innate biological differences among the perceived races. Early in the 20th century, Boas led the anthropological challenge to racist thinking.^24^

Later, Firth wrote in his book “Human Types,” “It is common to attribute ways of life and thought which we do not fully understand to racial differences.”^25^ This was followed by other criticisms of the very concept of human biological races. In 1964, Livingstone explained that genetic traits can often be discordant and “if two genes vary discordantly, the races set up on the basis of one do not describe the variability in the other.”^26^ In other words, genetic variation alone does not explain observable physical differences between populations, nor does it even support the notion of human biological races.

These critiques of biological race in humans was cemented by the work of Lewontin, who in 1972 set out to test whether conventional ideas about genetic racial differences were borne out in the data.^27^ He concluded from his studies of global populations that there was more genetic difference within purported human races than between them. As such, Lewontin declared, “Human racial classification is of no social value and is positively destructive of social and human relations. Since such racial classification is now seen to be of virtually no genetic or taxonomic significance either, no justification can be offered for its continuance.”^27^

Lewontin's scientific work challenging the notion of the existence of human biological races was preceded by and interwoven with other analyses and fundamental critiques of the global race narrative as well as the narrative of race within the United States and the lived experiences of African Americans.^28–33^ Du Bois' pioneering scholarship crystallized the argument that racism was a social process imprinted on U.S. politics and policies, and he consistently used his platforms to denounce racism and the misuse of science to justify and perpetuate it.^34,35^

Time and again, it has been demonstrated that there is no scientific evidence to support notions of separate human biological races, based on our knowledge of gene frequencies or variation within our species.^36,37^ Indeed, the very concept of biological race is derived from studying adaptation in nonhuman species.^38^ Comparison of human genetic variation to that of other large bodied mammals conclusively demonstrated that we have very little within species genetic variation; all modern humans are 99.9% identical in their genetic makeup. It might be legitimate to designate biological races within species such as Gray wolves, African wildebeest, or dog breeds, but not in modern humans. This is because human populations have a common African origin and have always maintained large amounts of gene flow (transfer of genetic material between populations), which unites our populations despite small amounts of adaptation to local environments.

Although the human species does not have and hierarchical races, we have fabricated and propagated the lie that it does. Even prominent modern-day geneticists can espouse this falsehood.^39^ The broader human genetics community, however, condemns belief in a racial hierarchy.^40^ Through the co-opting of biological taxonomy to categorize humans into hierarchical social groups called races, biological race and socially defined race have been conflated, leading to ongoing confusion about what race is and what it is not. Race in the context of humans is an entirely invented sociopolitical tool derived from a divisive ideology that ultimately has negative impacts on everyone in a racialized society.^41–43^

## Use of Race in Research and Health Care

Although there may well be growing consensus among scientists, scholars, and practitioners that race is not a biological or genetic category in humans,^44,45^ this has not prevented race from being incorporated in biomedical research and health care as if it were biologically deterministic. This is in part a byproduct of requirements by U.S. federal agencies to utilize the Office of Management and Budget (OMB) Census categories for maintaining, collecting, and reporting research data.^46^ It is also a function of the fact that researchers are members of society, and their development and evolution are influenced by existing and interrelated ideological, social, and political processes.^47^ Large-scale 21st century studies of geneticists and anthropologists—two groups whose disciplines have had substantial roles in both the manufacturing and dispelling of notions race and racial hierarchies—revealed complicated and differing perspectives on whether and how to use race in their research.^48,49^

The routine collection of racial data within health research has led to an assumption that race is somehow causative of disease.^50,51^ In addition to potentially influencing diagnostic decision-making processes, the use of racial and ethnic labels has also stimulated research in which a drug (BiDil) was licensed for use in a specific racialized group (“selfidentified blacks”).^52–54^ Although the complexities of the research and approval processes for BiDil are too detailed to include in this short article, it is sufficient to say that such racially marketed pharmaceutical products at the very least help reify the idea that racialized groups are biologically distinct.^55–57^

Within genetics and research specifically, there has been a tendency to assume that the minor genetic differences found to be associated with continental ancestry can justify the notion that racialized groups differ in their genetic composition in substantial ways that impact health. In response to this long-standing concern, the National Academies of Sciences, Engineering, and Medicine (NASEM) has established an *ad hoc* committee to develop recommendations for “best practices” regarding the use of race and other similar population descriptors in human genetics and genomics research.^58^ There is also a persistent racialization of diseases such as sickle cell disease and cystic fibrosis, which, although more prevalent in certain populations (people of recent African and European descent, respectively), are not absent from other populations.^59^

Given that these diseases are associated with known genetic variants that correlate with geographic ancestry, there is evident need to utilize our knowledge of this genetic variation in the study and management of these diseases. However, using sickle cell disease and cystic fibrosis as rationales for the continued focus on finding race-specific gene variants related to health diverts attention (and money) away from studies and interventions that might otherwise address the many social factors that are associated with racial health disparities (such as persistent inequities in income, education, and access to health care) in these and other diseases.

The continued use (and misuse) of race in research and health care does not mean that humans have biological races. It is incumbent on any biomedical researcher attempting to use socially defined race to reflect on the sociopolitical origins and continued employment of race to justify hierarchical thinking and actions. Similarly, social science researchers cannot simply ignore the fact that biological thinking is imbued with how societies conceptualize race and racial difference.

## Public Perspectives and Education on Race and Racism

Race-based practices notwithstanding, academics have largely embraced the notion that biological race does not exist in humans. However, perception of race as a biological category continues to influence public opinion, especially when coupled with essentialized thinking about genetic variation and difference.^60–62^ Biological racial thinking has merged with a sustained public and professional interest in that minor fraction of the genome that might be seen as marking genetic differences between purported races.^63^

**FIG. 1.**
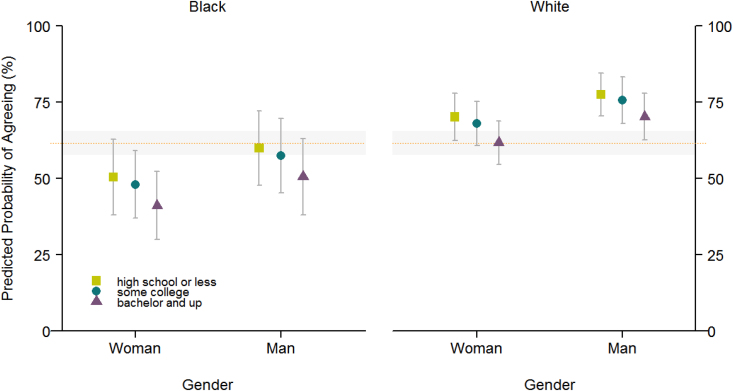
Levels of agreement on the existence of biological human races. Source: Royal et al (Unpublished data).

Public opinion surveys indicated that a majority of the general public in the United States believed that biological human races exist (Fig. 1) and that racial identity is determined largely by genetic information.^64,65^ The data also revealed that perceptions of race differed between populations by self-assigned race, ethnicity, age, socioeconomic status, and education level. Research has also demonstrated that although hierarchical racial thinking was not universal, certain elements of such thinking persist, especially in the attribution of sporting achievement among black athletes to innate natural gifts, whereas white athletes are credited with hard work and intelligence.^66,67^ Other surveys have similarly found that essentialized views of genetics as determining certain behavioral and personality traits play an influential role in the public understanding of race, especially in whites attributing multiple observable traits to genetics among blacks (an attribution not ascribed to whites by whites).^68,69^

In parallel to (and sometimes overlapping) the professional employment of race in genomics research, the early 21st century has seen a great deal of public interest in commercial genetic ancestry testing services.^70–72^ Although this has raised concerns that such interest reflects a growing reversion back to notions of biological thinking about racial identification and difference, recent studies reveal that members of the public have a nuanced appreciation of such testing. Indeed, interviews with members of the public suggest that genetic ancestry plays into notions of self-identity but does not determine these ideas.

As Roth and Ivemark have suggested, genetic ancestry testing consumers “pick the truths they want from their genetic options” rather than accepting the test results as determining identity.^73^ These findings suggest that genetics is being employed by consumers as something to add to their notion of identity, a social and genetic mix of identity. Genetic ancestry testing appeals to often highly contradictory ideas about community as natural identities and very personalized desires to find oneself in a highly mobile world.^74–76^

False beliefs about genetic variation can negatively affect views of and interactions with other racialized groups.^77,78^ The need to dismantle racial ideology is more urgent now than ever. The national Truth, Racial Healing & Transformation (TRHT) movement is a uniquely holistic effort that regards the dismantling of racial ideology as integral to the narrative change process in the movement's strategy for addressing racism.^79^ This approach is aligned with research by Donovan, which indicates that accurate scientific education on human genetic variation can be useful in reducing the prevalence of racial bias.^80^ More quantitative research on pedagogical outcomes of genetics education of this sort would be greatly beneficial. Other outstanding efforts to increase public education about the connections among race, science, and society include the 2003 documentary, “Race: the Power of an Illusion,” and the 2006 museum exhibit, “Race: Are we so different?.”^81,82^ On a global scale, the United Nations Educational, Scientific, and Cultural Organization (UNESCO) has issued multiple statements on race and racism.^83^ The first, published in 1950, asserted that race is not a biological reality but a myth that has caused suffering.

## Conclusion

Although there are no biological human races, there are racialized human groups, each of which has both a shared cultural identity and a range of cultural identities within. This brief is not advocating for the negation of those invaluable identities or for so-called colorblindness. Instead, it is a call for eliminating racism. Truth-telling about the origins of racial thinking, the inseparable foundation of racial thinking in racism, and the ongoing individual and collective damage created by racism is vital for narrative change. The practice of racism produces and prolongs the illusion of race.^5^ We cannot separate race from racism and should not continue to blur social and biological definitions of race in our personal, professional, and public lives. We need to move forward with a clear understanding and a more accurate and complete narrative. Only then can we experience full and lasting liberation from the shackles of ignorance, confusion, and devastation imposed by racism and the myth of race. Some important takeaways are provided in Box 1.

## Notes

Box 1. Key Points

1. Racism preceded the idea that biological human races exist.
2. Socially defined race was created to reflect and reinforce social hierarchy.
3. Biological race is determined by the amount of genetic variation between and within subgroups of a species, or by whether any subgroup within a species can be considered an evolutionary distinct lineage. Some species can be said to have biological races, but our species, anatomically modern humans, does not.
4. Research has repeatedly shown that genetic variation is greater within human populations than between them.
5. Genetic variation in the human species exhibits gradients of differentiation across geography, not subdivisions into homogeneous, discontinuous units.
6. The observed physical traits that are commonly used to define or identify socially defined racial groups (especially skin color) are not necessarily linked to each other or to health, disease, or other unobservable traits.
7. Assumptions that racial identity labels are markers for clinically relevant genetic differences have led to countless errors in attributing health disparities and inequities to perceived innate biological differences, without any evidence.
8. The focus on trying to find genetic etiologies for common health disparities diverts resources away from addressing the social inequities that underlie these disparities.
9. Racial inequities and disparities can only be fully addressed by the elimination of racism through a collective change in thinking about race and racialized groups.
10. Policy makers and other change agents are critical to the dismantling of ideas and systems of race and racism.
